# Dibutyl Phthalate Adsorbed on Multiwalled Carbon Nanotubes Causes Fetal Developmental Toxicity in Balb/C Mice

**DOI:** 10.3390/toxics11070565

**Published:** 2023-06-29

**Authors:** Yujie Qin, Suli He, Haiyan Peng, Xin Ye, Hongmao Zhang, Shumao Ding

**Affiliations:** 1 Hubei Key Laboratory of Genetic Regulation and Integrative Biology, School of Life Sciences, Central China Normal University, Wuhan 430079, China; qyj5459@163.com (Y.Q.); hesuli0093@163.com (S.H.); phy1553882@163.com (H.P.); 2 Liquor Marking Biological Technology and Application of Key Laboratory of Sichuan Province, College of Bioengineering, Sichuan University of Science & Engineering, Yibin 644000, China

**Keywords:** multiwalled carbon nanotubes, dibutyl phthalate, compound exposure, mice, reproductive toxicity

## Abstract

This study investigated whether using multiwalled carbon nanotubes (MWCNTs) as a carrier for dibutyl phthalate (DBP) could delay the degradation rate of DBP in mice and increase its estrogen-like interference effect. Pregnant Balb/C mice were divided into four groups and exposed to different treatments via tail-vein injection every 3 days until gestational day 20. The female and male mice were then sacrificed for toxicological study. The results showed that the combination of MWCNTs and DBP resulted in a higher fetal mortality rate than if the mice were exposed to MWCNTs or DBP alone. H&E staining showed that the estrous period of the exposed mice was delayed, the development of oocytes was blocked in the combination group, the number of spermatogenic cells decreased, and the quality of sperm decreased. Our experiment showed that the expression levels of the genes involved in sex hormone synthesis in the testis and ovaries were significantly increased after combined treatment compared with the MWCNT group (*p* < 0.01). The study suggests that DBP degradation is delayed when absorbed on MWCNTs, which increases its estrogen-like interference and interferes with fetal development, ultimately leading to increased fetal mortality.

## 1. Introduction

Carbon nanotubes (CNTs) are a kind of carbon nanomaterial with a layered hollow structure. They are tubes made of single or multilayer graphite cylinders along with the coaxial layer. It is estimated that the global annual production of carbon nanotubes will increase from 5300–5720 tons in 2015 to 10,500–12,000 tons in 2020 [1]. Along with the production and application of CNTs, they are correspondingly released into the environment. Concentrations of CNTs in environmental sediments are predicted to range from 0.0004 to 1260 μg/kg [2]. According to the number of graphite layers, carbon nanotubes can be divided into single-walled carbon nanotubes (SWCNTs) and multiwalled carbon nanotubes (MWCNTs) [3]. Due to their superior mechanical, electrical, and chemical properties [4], CNTs have been successfully used in many fields such as plastics, battery electrodes, water purification systems [5], and adhesives. Recently, CNTs have also been shown to have good prospects for use in the fields of drug delivery, tumor therapy, and biomedical materials [6,7,8].

Due to the quantum size effect, surface effect, surface adsorption, and other reasons, nanomaterials exhibit many special biological activities in biological organisms and may have various unknown effects on the functions of these organisms. The potential harm of nanomaterials to human health and the ecological environment has attracted the attention of many scholars [9,10,11]. Although CNTs are composed of graphite that is considered nontoxic or of low toxicity, studies have shown that the toxicity of CNTs is much greater than that of graphite powder. Because of their extremely small particle size, CNTs may enter the body through the pulmonary blood barrier or the skin via simple diffusion or penetration [12]. Sridharan demonstrated that MWCNTs are carcinogenic in vitro [13]. The above experiments prove that CNTs can easily pass through cell membranes by simple osmosis. Adriana found that MWCNTs can quickly enter the body, deposit in the lung and brain, and induce macrophages to recruit amyloid deposits [14]. In addition, there is evidence that CNTs can cross the blood–testis barrier of mice when administered intragastrically and cause damage to the testis [15]. Studies have shown that MWCNT exposure in pregnant mice can be toxic to their offspring [16].

Phthalates (PAEs) are a class of synthetic organic compounds that are used as plasticizers and softeners in industrial and agricultural products such as plastic film and lubricants [17]. PAEs and plastic components are held together by hydrogen bonds or van der Waals forces, which have weak bond energy; hence, PAEs are easily released into the environment [18]. Studies have shown that PAEs can be widely detected in water, soil, and air. In agricultural soils in different regions of China, the total concentrations of PAEs mainly ranged from 0 to 35.4 mg/kg [19]. PAEs can cause endocrine disorders by binding hormone receptors in the body [20]. Dibutyl phthalate (DBP) is one of the most commonly used phthalates and is often found in contaminated water, plants, soil, sediment, air, food, and even human body fluids [21]. In the Yangtze River Delta (YRD) region, DBP was the dominant PAE congener in water, accounting for 50.6% of the sum concentrations of nine PAEs [22]. Zhu found that the detection rate of DBP metabolites in urine was 99.1% [23]. Animal studies have shown significant concentrations of DBP in venous blood, serum, and tissues [24,25].

Animal experimental studies have shown that DBP has “hormonal activity”, resulting in reproductive toxicity [26]. Shinkut found that DBP can induce oxidative stress, impact sperm DNA integrity, and increase testicular oxidative stress biomarkers [27]. Liu found that DBP exposure caused dose-dependent effects on folliculogenesis and gene expression in mouse [28]. Pregnant women, as a unique population, are particularly sensitive to various environmental pollutants. Epidemiological investigations have found the presence of at least five phthalate metabolites in the urine of pregnant women, and various PAEs such as DiBP, DBP, and DEHP have potential health threats to pregnant women, and maternal exposure to PAEs is related to housing characteristics (type of housing, type of household fuel, and installation of kitchen exhaust system) [29,30].

Carbon nanotubes can easily enter cells through penetration or phagocytosis. Although CNTs are cytotoxic, some of their properties make them very attractive for medical applications. Due to the highly hydrophobic nature of CNTs, they can easily pass through biological membranes without damaging the membrane, and accumulate in cell compartments, making them an innovative drug delivery device [31,32].

The hollow lumen of carbon nanotubes can adsorb drugs, which can be used as a carrier to deliver drugs to cells or tissues. The main mechanism for MWCNTs to adsorb drugs is the π–π bond between MWCNTs and organic molecules. The carbon atoms in the tubular structure of MWCNTs form highly delocalized large π bonds, and these π electrons can interact with other π electron-containing compounds through π–π interactions [33,34,35]. DBP is a compound based on an aromatic benzene ring, which has a large π bond formed by six unhybridized p-orbitals. In the environment, they are prone to forming compounds. Studies have shown that a new type of magnetic multi-template molecularly imprinted polymer based on multiwalled carbon nanotubes can adsorb DBP in environmental water samples [2]. If DBP is adsorbed on MWCNTs in the environment, they will enter the animal body like a nanotube-drug carrier adsorbed DBP. Carbon nanotubes act as a virtual protective umbrella for DBP and, thus, delay the degradation of DBP in the body. In this way, the endocrine-disrupting effect of DBP will be prolonged, and the toxicity will be increased. When MWCNTs adsorb DBP and then enter tissue, DBP is carried into the cells. In this way, a “hormone-receptor regulation” mode may appear, which can affect the expression of many genes in cells resulting in metabolic disorders in these cells. In this study, female and male Balb/C mice were individually and jointly exposed to MWCNTs and DBP, to explore whether exposure to MWCNTs carrying DBP can increase the endocrine-disrupting effect of DBP, as well as affect the fetal development in mice. Our research has important scientific significance for evaluating the risk and safety of exposure to MWCNTs combined with DBP.

## 2. Materials and Methods

### 2.1. Animals

All experimental guidelines were approved by the Institutional Animal Care and Use Committee, Ethics Review Committee for Life Science Study of Central China Normal University with the reference number CCNU-IACUC-2018-005. Eighty (5–6 week old) female and male Balb/C mice were purchased from the Center for Laboratory Animal Administration, Center for Disease Control and Prevention, Hubei Province (Wuhan, China). A total of 40 female Balb/C mice were randomly divided into four groups, 40 male Balb/C mice were also divided into four groups (n = 10/group): control group; 10 mg/kg MWCNT group; 2.15 mg/kg DBP group; MWCNT + DBP group. The animals were housed in standard environmental conditions (12 h light-dark cycle, 50–70% humidity, and 20–25 °C). The mice were provided with adequate food and water.

### 2.2. Reagents and Kits

Reagents: DBP was purchased from Sigma-Aldrich, USA. MWCNTs (outer diameter less than 8 nm, length 0.5–2 μm) were purchased from Shanghai Aladdin Biochemical Technology Co., Ltd. (Shanghai, China, File S1).

Kits: The DBP ELISA kit was purchased from Prime Bio Tek. BCA Protein Quantification Kit was purchased from Vazyme Biotech Co., Ltd. (Nanjing, China). GSH detection kits were purchased from Nanjing Jiancheng Bioengineering Institute.

### 2.3. Preparation and Exposure Conditions

#### 2.3.1. Preparation of Mixed Reagents

We prepared 100 mL of 12.5 mg/L DBP solution in physiological saline, and then incorporated 50 mg of MWCNTs. After shaking at 25 °C, 150 r/min for 8 h, and centrifuging at 3000 rpm for 30 min, we then filtered with a membrane and detected the remaining DBP concentration with a DBP ELISA kit. The results of the adsorption experiment found that the saturated adsorption capacity of MWCNTs for DBP was 215 mg/g.

#### 2.3.2. Exposure Conditions

We used physiological saline as the solvent to prepare the four exposure solutions. Tail vein injections were given to mice every 3 days. Before each tail vein injection, the exposure solutions were ultrasonically oscillated for 30 min. The body weight of each mouse was recorded daily during the exposure period. After 2 weeks of exposure, the male and female mice were placed in a cage to mate at a ratio of 1:1 for 10 days. During the mating period, the female mice were checked to find the vaginal plug indicating the start of pregnancy. The exposure protocol was maintained during this period, and the mice were sacrificed on the 20th day after the female became pregnant.

### 2.4. Fetal Development Statistics

The gestation period of female mice is generally 18–22 days. After 20 days, the fetus is mature, and we can observe the development of the fetal very well. On the 20th day of pregnancy, the pregnant mice were sacrificed, and the required organs and fetus were removed. We recorded the number of live births per litter, and the mortality of the fetus was recorded for each group.

### 2.5. Sperm Quality Test

#### 2.5.1. Sperm Density Test

We dropped 10 μL of sperm suspension on a cell counting plate and immediately observed it under an ordinary optical microscope. The calculation to determine sperm density is as follows:

Sperm density = the sum of the number of sperm in 5 middle squares/80 × 400 × 10,000 × dilution multiple.

#### 2.5.2. Sperm Deformity Rate Test

We filtered the sperm suspension using lens cleaning paper. Then, we added a 1% eosin solution for staining. We then examined 300 sperm from each mouse under microscope to determine the number of abnormal sperm and calculate the sperm deformity rate.

### 2.6. Histological Assay of the Ovaries and Testes

Hematoxylin and eosin (H&E) staining was used to detect pathological changes in the ovarian and testicular tissues of mice from each group. We immersed the excised ovaries and testes in 4% paraformaldehyde solution for 24 h and then dehydrated them in ethanol. Distal sections were embedded in paraffin, cut into 5 μm sections, and stained with H&E. We examined the stained tissue using an upright differential interference microscope (OLYMPUS, Tokyo, Japan) to look for any morphological changes.

### 2.7. GSH and MDA

The ovaries and testes of the mice were excised and weighed, and then homogenized in 10 mL/g ice-cold 1× phosphate-buffered saline (PBS, pH = 7.5). The homogenate was centrifuged at 10,000 rpm for 10 min at 4 °C. The supernatant was collected for immediate measurement of MDA and GSH levels. MDA, an indicator of lipid peroxidation, was determined using the thiobarbituric acid method. GSH detection kits from Nanjing Jiancheng Bioengineering Institute were used to determine GSH levels.

### 2.8. Mass Spectrometry Analysis of Proteins in Serum and Testis

#### 2.8.1. Mass Spectrometry Analysis of Protein in Female and Male Mouse Serum

The serum was mixed with 50 mM NH_4_HCO_3_ and centrifuged to obtain the supernatant. We used the BCA method for protein quantification. After that, we added acetone at 4 °C and left it overnight, before centrifuging and discarding the supernatant. Precooled methanol was added and centrifuged to remove the methanol. To this, we added 50 mM NH_4_HCO_3_ to redissolve the dried precipitate. Next, we added 10 mM DTT for 30 min reduction, followed by 15 mM IAM for 30 min alkylation. We then added 2 μg of trypsin sequencing grade to digest for 18 h. Finally, we added formic acid to the sample to stop the enzymatic hydrolysis reaction.

Before loading the mass spectrometer, we used 0.1% formic acid solution to reconstitute. We used an Exactive™ Plus Orbitrap high-resolution mass spectrometer to load and analyze peptide samples. The mass spectrometer used a data-dependent acquisition mode (DDA). The mass-to-charge ratio scanning range of the first-order spectrum was 350–2000, and the AGC is 3 × 10^6^ ions. The fragmentation mode was HCD, the resolution of the first spectrum was 70,000, and the resolution of the second spectrum was 7500. We selected the top 20 ions for secondary spectrum detection, the isolation window was 1.8 *m/z*, the dynamic exclusion duration was 40 s, and the lowest primary spectrum signal intensity that triggered the secondary spectrum was 5000.

#### 2.8.2. Mass Spectrometry Analysis of Proteins in Testis

First, we added the testicular tissue to the RIPA lysis buffer, ground it on ice, and incubated it for 1 h. The tissue solution was centrifuged to obtain the supernatant, and the BCA method was used for protein quantification, as described in Section 2.8.1.

### 2.9. The Related Genes Test

Total RNA extraction from ovaries and testes was performed using Trizol reagent (Thermo Fisher Scientific, Waltham, MA, USA) according to the manufacturer’s instructions. Total RNA was used for cDNA synthesis using the FastKing gDNA Dispelling RT SuperMix (Beijing TIANGEN Biotech Co., Ltd., Beijing, China) according to the manufacturer’s instructions. qRT-PCR analysis was performed using β-actin expression as the internal standard to normalize the amount of cDNA. Transcript levels of genes were quantified by qRT-PCR using the CFX96 Touch detection system. Amplifications were performed using ChamQ Universal SYBR qPCR Master Mix (Vazyme Biotech Co., Ltd.), and the gene-specific primers of the genes are shown in Table 1. The conditions were 95 °C for 30 s, 40 cycles at 95 °C for 10 s, and 60 °C for 30 s, with the last step using the instrument’s default dissolution curve acquisition program. All qRT-PCR expression assays were performed and analyzed at least three times in independent experiments. The relative mRNA level (normalized to the level of β-actin gene) of each specific transcript was determined with the Bio-Rad software and calculated using the 2^−ΔΔCT^ method.

### 2.10. Statistical Analysis

Data are presented as the mean ± SEM. Statistical graphs were generated using GraphPad Prism 7.0 (San Diego, CA, USA). A one-way ANOVA combined with *t*-tests between groups was used to determine the significance of differences. A *p*-value < 0.05 was considered significant, and a *p*-value < 0.01 was considered extremely significant. Data analyses were carried out using SPSS ver27 (SPSS, Chicago, IL, USA).

Mass spectrometry (MS) data were processed using the commercially available software, Proteome discoverer 2.0. All searches were performed against the UniProt protein database subset of mouse protein. The retrieval and analysis of protein data used Origin 2019 software and the DAVID Bioinformatics Resources 6.8 website (https://david.ncifcrf.gov/ accessed on 8 May 2023). Proteins with a 2.0-fold change and a *p*-value < 0.05 (*t*-test) were determined to be differentially expressed proteins, and results were presented in volcano plot form using GraphPad Prism 7.0.

## 3. Results

### 3.1. Fetal Development Statistics

The number of live births per litter, and the stillbirth rate for each group (Table 2) showed that there was no significant difference in the number of live births per litter between the groups. However, the fetal mortality rate for the combined exposure group was significantly higher than that of the control group (*p* < 0.05).

### 3.2. Sperm Quality Test

#### 3.2.1. Sperm Density Test

**Figure 1:**
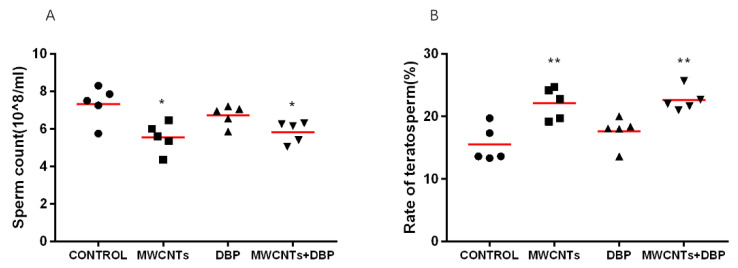
Sperm quality: (**A**) sperm density; (**B**) sperm deformity rate. * *p* < 0.05, compared with the control group; ** *p* < 0.01, compared with the control group.

Figure 1A shows the sperm count of the male mice from each group. Compared with the control group, the sperm counts for the mice in the MWCNT only group and the combined exposure group were significantly decreased (*p* < 0.05). The sperm count of the mice in the DBP group did decrease, but this was not significant.

#### 3.2.2. Sperm Deformity Rate Test

The sperm count and deformity index are key to assessing male fertility. The deformity rate of sperm reflects the degree of damage to the sperm of mice caused by exposure. The results (Figure 1B) showed that, compared with the control group, the sperm deformity rate in the MWCNT only group and the combined exposure group was significantly increased (*p* < 0.01), while the sperm deformity rate for the DBP group demonstrated no significant difference.

### 3.3. Histological Observation of the Ovaries and Testis

#### 3.3.1. Histological Observation of the Ovaries

**Figure 2:**
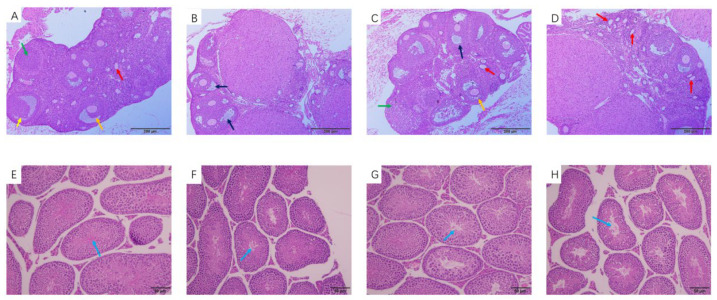
Hematoxylin and eosin staining of ovary tissue (magnification: ×100). (**A**) Control group; (**B**) MWCNT group; (**C**) DBP group; (**D**) MWCNT + DBP group. Hematoxylin and eosin staining of testicular tissue (magnification: ×200): (**E**) control group; (**F**) MWCNT group; (**G**) DBP group; (**H**) MWCNT + DBP group. Red arrows indicate primary follicles, yellow and orange arrows indicate secondary follicles, green arrows indicate corpus luteum, black arrows indicate atretic follicles, and blue arrows indicate spermatogenic cells.

Observation of the tissue slices showed pathological changes in the ovaries of the female mice (Figure 2A–D). The number of primary follicles in the ovaries of the MWCNT exposure group decreased compared with the control group, while the number of atretic follicles increased (Figure 2B). The number and type of follicles in the ovaries of the DBP group was not significantly different from that of the control group, but the number of atretic follicles increased (Figure 2C). The number of primary follicles in the ovarian tissue of the combined exposure group increased (Figure 2D).

#### 3.3.2. Histological Observation of the Testis

Figure 2E–H show pathological changes in the testes of mice. Compared with the control group, the number of spermatogenic cells in the testes of the male mice in the MWCNT group (Figure 2F) and the DBP group (Figure 2G) decreased, and there was slight damage. Figure 2H shows that the number of spermatogenic cells in the testes of the mice in the combined exposure group was reduced, the cell arrangement was disordered, and the damage was more serious.

### 3.4. Effect of Oxidative Stress

#### 3.4.1. Oxidative Stress in the Ovaries

**Figure 3:**
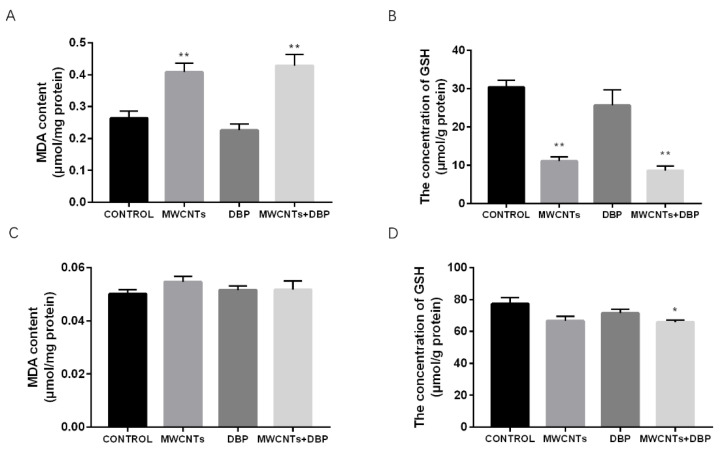
The effect of oxidative stress in ovaries (**A**,**B**) and testis (**C**,**D**). Level of MDA (**A**) and GSH (**B**) in the ovaries (n = 10); level of MDA (**C**) and GSH (**D**) in the testis (n = 10). * *p* < 0.05, compared with the control group; ** *p* < 0.01, compared with the control group.

The levels of MDA and GSH in the ovaries of the different groups are shown in Figure 3A,B. Compared with the control group, the content of MDA in the ovaries of the MWCNT group and the combined exposure group increased significantly (*p* < 0.01), while the content of GSH in the ovaries decreased significantly (*p* < 0.01). 

#### 3.4.2. Oxidative Stress in the Testis

The levels of MDA and GSH in the testis of the different groups are shown in Figure 3C,D. Compared with the control group, the testicular MDA levels of each experimental group changed; however, this was not significant. The testicular GSH levels of the combined exposure group were significantly lower than those of the control group (*p* < 0.05).

### 3.5. Mass Spectrometry Analysis of Protein in Female Mouse Serum

#### 3.5.1. Venn Analysis

**Figure 4:**
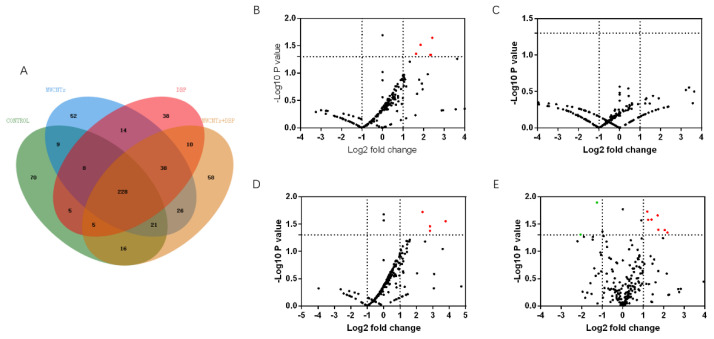
Venn analysis and Volcano plots analysis of serum protein in female mice. (**A**) The results of Venn analysis of serum protein profile of female mice; the volcano plots of the differential expression of proteins in female serum protein: (**B**) control vs. MWCNTs; (**C**) control vs. DBP; (**D**) control vs. MWCNTs + DBP; (**E**) MWCNTs vs. MWCNTs + DBP. In the volcano plots, red represents upregulated proteins, green represents downregulated proteins, and black represents non-differentially expressed proteins.

We used mass spectrometry to analyze the protein content in the serum of female mice from each group. Venn analysis (Figure 4A) showed that 228 proteins were co-expressed in the serum of the four groups of female mice. The number of proteins specifically expressed in the control group, the MWCNT group, the DBP group, and the combined exposure group was 70, 52, 38, and 58, respectively.

#### 3.5.2. Volcano Analysis

The results of the volcano analysis of the differential expression of serum proteins co-expressed by female mice in each group are shown in Figure 4B–E. Red represents significantly upregulated protein, and green represents significantly downregulated protein. The results show that, compared with the control group, eight serum proteins were significantly upregulated in the MWCNT group, and seven were significantly upregulated in the combined exposure group (*p* < 0.05, FC > 2). The DBP group had no significantly differentially expressed proteins. Compared with the MWCNT group, 10 proteins in the combined exposure group were significantly upregulated (*p* < 0.05, FC > 2), and two proteins were significantly downregulated (*p* < 0.05, FC < $\frac{1}{2}$).

### 3.6. Mass Spectrometry Analysis of Protein in Male Mice Serum

#### 3.6.1. Venn Analysis

**Figure 5:**
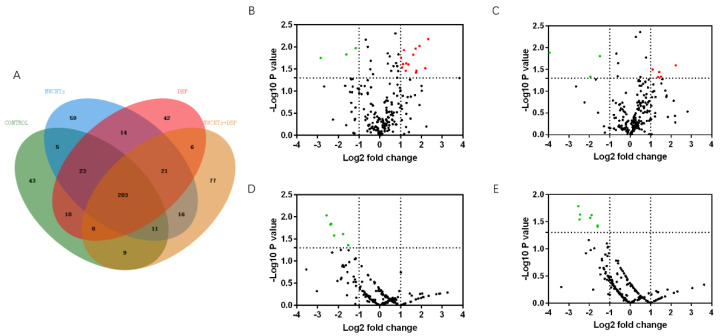
Venn analysis and Volcano plots analysis of serum protein in male mice. (**A**) The results of Venn analysis; the volcano plots of the differential expression of protein in male serum: (**B**) control vs. MWCNTs; (**C**) control vs. DBP; (**D**) control vs. MWCNTs + DBP; (**E**) MWCNTs vs. MWCNTs + DBP. Red represents upregulated proteins, green represents downregulated proteins, and black represents non-differentially expressed proteins.

We used mass spectrometry to analyze the protein content in the serum of male mice from each group. Venn analysis (Figure 5A) showed that 203 proteins were co-expressed in the serum of the four groups of male mice. The number of proteins specifically expressed in the control group, the MWCNT group, the DBP group, and the combined exposure group was 43, 59, 42, and 77, respectively.

#### 3.6.2. Volcano Analysis

The results of the volcano analysis of the differential expression of serum proteins co-expressed by male mice in each group are shown in Figure 5B–E. Red represents significantly upregulated protein, and green represents significantly downregulated protein. Compared with the control group, 14 serum proteins were significantly upregulated in the MWCNT group, and five serum proteins were significantly upregulated in the DBP group (*p* < 0.05, FC > 2). Three proteins were significantly downregulated in both these groups (*p* < 0.05, FC < $\frac{1}{2}$). Compared with the control group, six proteins in the combined exposure group were significantly downregulated (*p* < 0.05, FC < $\frac{1}{2}$). Compared with the MWCNT group, seven proteins in the combined exposure group were significantly downregulated (*p* < 0.05, FC < $\frac{1}{2}$). 

### 3.7. Mass Spectrometry Analysis of Proteins in Testis

#### 3.7.1. Venn Analysis

**Figure 6:**
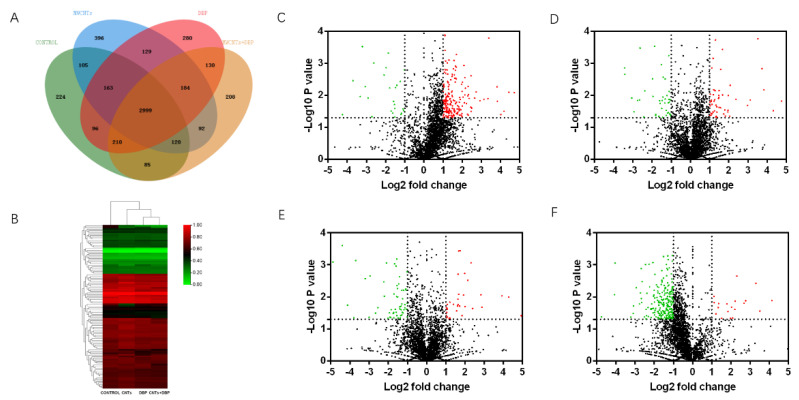
Venn analysis and Volcano plots analysis of proteins in testis. (**A**) The results of Venn analysis of testis protein; (**B**) heatmap of differential proteins in testis; the volcano plots of the differential expression of protein in testis: (**C**) control vs. MWCNTs; (**D**) control vs. DBP; (**E**) control vs. MWCNTs + DBP; (**F**) MWCNTs vs. MWCNTs + DBP. Red represents upregulated proteins, green represents downregulated proteins, and black represents non-differentially expressed proteins.

We used mass spectrometry to analyze the protein content in the testis of male mice from each group. Venn analysis (Figure 6A) showed that 2999 proteins were co-expressed in the testis of the four groups of male mice. The number of proteins specifically expressed in the control group, the MWCNT group, the DBP group, and the combined exposure group was 224, 396, 280, and 208, respectively.

#### 3.7.2. Heatmap and Volcano Plots Analysis

The result of the heatmap analysis of the differential expression of testicular proteins co-expressed by male mice from each group is shown in Figure 6B. The volcano analysis results are shown in Figure 6C–F. Red represents significantly upregulated protein, and green represents significantly downregulated protein. Compared with the control group, 194 serum proteins in the MWCNT group were significantly upregulated (*p* < 0.05, FC > 2), and 27 proteins were significantly downregulated (*p* < 0.05, FC < $\frac{1}{2}$). In the DBP group, 51 serum proteins were significantly upregulated (*p* < 0.05, FC > 2), and 40 proteins were significantly downregulated (*p* < 0.05, FC < $\frac{1}{2}$). In the combined exposure group, 34 serum proteins were significantly upregulated (*p* < 0.05, FC > 2), and 59 proteins were significantly downregulated (*p* < 0.05, FC < $\frac{1}{2}$). Compared with the MWCNT group, 20 serum proteins in the combined exposure group were significantly upregulated (*p* < 0.05, FC > 2), and 229 proteins were significantly downregulated (*p* < 0.05, FC < $\frac{1}{2}$).

#### 3.7.3. Gene Ontology Enrichment Analysis

**Figure 7:**
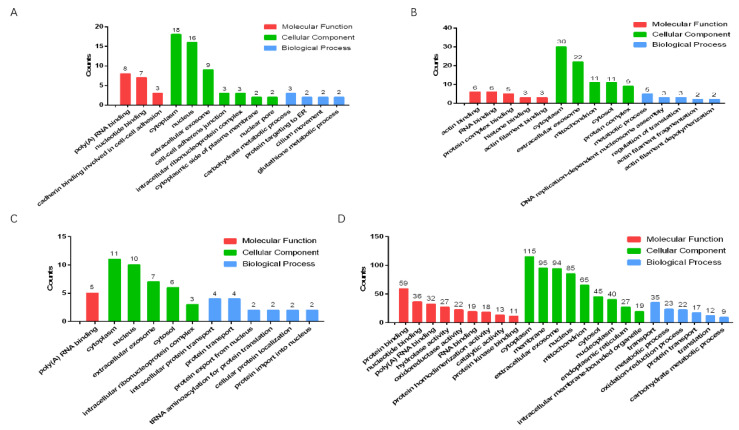
Gene Ontology analysis of the proteins in testis. (**A**) Significantly upregulated proteins in the control group vs. the MWCNT + DBP group; (**B**) significantly downregulated proteins in the control group vs. the MWCNT + DBP group; (**C**) significantly upregulated proteins in the MWCNT group vs. the MWCNT + DBP group; (**D**) significantly downregulated proteins in the MWCNT group vs. the MWCNT + DBP group. BP: biological process; CC: cellular component; MF: molecular function.

We used Gene Ontology (GO) to analyze the significantly upregulated and downregulated proteins in the testes of male mice in the combined exposure group compared with the control group and the MWCNT group (Figure 7A–D). The results showed that, compared with the control group, most of the significantly upregulated proteins in the combined exposure group were related to the cytoplasm (Figure 7A), while most significantly downregulated proteins were related to cytoplasm proteins (Figure 7B). Compared with the MWCNT group, the significantly upregulated proteins in the combined exposure group had an increased proportion of proteins involved in biological processes (Figure 7C), and the significantly downregulated proteins were mostly related to protein binding and membranes (Figure 7D).

### 3.8. The Related Genes Test

#### 3.8.1. The Results of Steroid Biosynthesis Testing in the Ovaries

**Figure 8:**
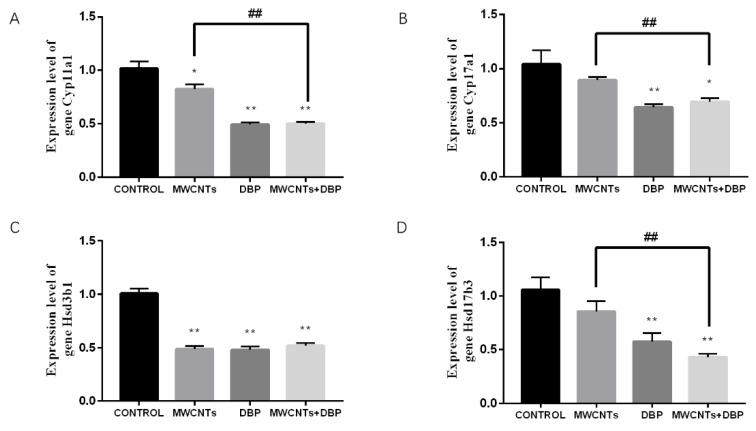
The results of qRT-PCR analysis of steroid biosynthesis in the ovaries. (**A**) The expression level of the gene *Cyp11a1*. (**B**) The expression level of the gene *Cyp17a1*. (**C**) The expression level of the gene *Hsd3b1*. (**D**) The expression level of the gene *Hsd17b3*. * *p* < 0.05, compared with the control group; ** *p* < 0.01, compared with the control group; ^##^ *p* < 0.01, compared with the MWCNT group.

The qRT-PCR analysis results (Figure 8) showed that, compared with the control group, expression of the *Cyp11a1* gene in the ovaries of the MWCNT group, the DBP group, and the combined exposure group was significantly reduced. Compared with the MWCNT group, expression of the *Cyp11a1* gene in the ovaries of the combined exposure group was significantly reduced (*p* < 0.01) (Figure 8A). Compared with the control group, expression of the *Cyp17a1* gene in the ovaries of the DBP group and the combined exposure group was significantly reduced. Compared with the MWCNT group, the expression of the *Cyp17a1* gene in the ovaries of the combined exposure group was significantly different (*p* < 0.01) (Figure 8B). Compared with the control group, the expression of the *Hsd3b1* gene in the ovary of the other experimental groups was significantly different (*p* < 0.01) (Figure 8C). Compared with the control group, expression of the *Hsd17b3* gene in the ovaries of the DBP group and the combined exposure group was extremely significantly reduced (*p* < 0.01). The *Hsd17b3* gene expression in the ovaries of the combined exposure group was significantly different from that of the MWCNT group (*p* < 0.01) (Figure 8D).

#### 3.8.2. The Results of Steroid Biosynthesis Testing in the Testis

**Figure 9:**
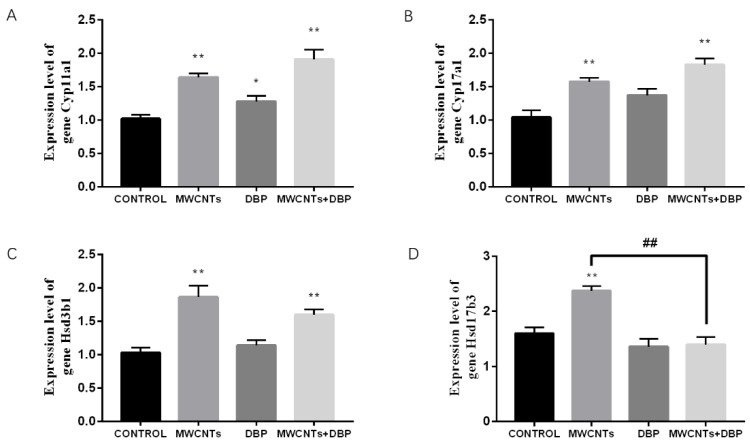
The results of qRT-PCR analysis of steroid biosynthesis in the testis. (**A**) The expression level of the gene *Cyp11a1*. (**B**) The expression level of the gene *Cyp17a1*. (**C**) The expression level of the gene *Hsd3b1*. (**D**) The expression level of the gene *Hsd17b3*. * *p* < 0.05, compared with the control group; ** *p* < 0.01, compared with the control group; ^##^ *p* < 0.01, compared with the MWCNTs group.

The qRT-PCR analysis results (Figure 9) showed that, compared with the control group, *Cyp11a1* gene expression in the testes of the MWCNT group, the DBP group, and the combined exposure group increased significantly (Figure 9A). Compared with the control group, *Cyp17a1* gene expression in the testis of the MWCNT group and the combined exposure group increased significantly (*p* < 0.01) (Figure 9B). Compared with the control group, *Hsd3b1* gene expression in the testes of the MWCNT group and the combined exposure group increased significantly (*p* < 0.01) (Figure 9C). Compared with the control group, the *Hsd17b3* gene expression in the testis of the MWCNT group was significantly increased (*p* < 0.01). Compared with the MWCNT group, the *Hsd17b3* gene expression in the testis of the combined exposure group was significantly different (*p* < 0.01) (Figure 9D).

## 4. Discussion

The numbers of live births and stillbirths are commonly used as indicators when evaluating reproductive and developmental toxicity. In this study, the fetal mortality rate for the combined exposure group increased significantly. We found that the combination of MWCNTs and DBP had no significant effect on the total number of offspring. We can speculate that the combined treatment does not affect the formation of the fetus, but affects the development of progeny and leads to increased fetal mortality.

We explored the reasons for the increase in fetal mortality at the individual level, organ level, and molecular level of the female and male parent mice.

Sperm quality is affected by a series of factors including lifestyle (such as smoking and drinking), physical health (such as obesity, mental health, and sleep disorders), and the environment (such as pesticides and phthalates) [36]. Sperm count and sperm deformity rate are important indicators for evaluating sperm quality, and male fertility. We found that both the MWCNT group and the combined exposure group had lower sperm counts and higher deformity rates compared to control; however, there was no significant difference between the two groups for these indicators.

Female reproductive potential can be determined by the number of ovarian follicles [37]. In the early stage of the estrus cycle of female mice, we found that the combination of MWCNTs and DBP could significantly delay the estrus of female mice. Histopathological observations showed that the number of primitive follicles in the ovaries of the combined treatment group was relatively higher than that of the control group, indicating that the combination of MWCNTs and DBP delayed the development of follicles in the ovaries of female mice.

Histopathological observation of mice testis showed that the combined treatment of MWCNTs and DBP resulted in a reduced number of spermatogenic cells in the testis, and their arrangement was disordered, resulting in reproductive injury.

Studies have found that high concentrations of DBP can affect sperm quality in mice, while low concentrations of DBP have little effect [38]. In this study, we used the low concentration of DBP, which is the reason why we could not have observable pathological damage to the ovary and testis. We, therefore, concluded that the pathological lesions observed in the ovary and testis caused by the combined treatment were primarily caused by the MWCNTs.

In healthy organisms, there is a dynamic balance between the oxidative system and the antioxidant system. When foreign substances enter the body, this balance may be upset; in this case, the body will try to restore the balance through the oxidative stress response. When the damage caused by toxic substances exceeds the body’s antioxidant capacity, the balance is destroyed, and the levels of MDA increase and redox substances such as GSH decrease, leading to oxidative damage [39]. Damage to the male reproductive system caused by oxidative stress is an important factor in most cases of male infertility [40]. The testis is susceptible to the influence of external substances, and its self-regulation ability is weak. Exposure to certain toxins increases the oxidative stress levels in the testis and reduces male fertility [41].

Through analyzing the results for the related indicators of oxidative damage, we found that MWCNTs played a major role in the oxidative damage of ovarian tissue caused by the combined treatment. However, in testicular tissue, the degree of oxidative damage caused by the combined treatment was not obvious. These results proved at the molecular level that the degradation of DBP in mice did not increase the oxidative damage to mice.

We analyzed the serum differential proteins of female mice by mass spectrometry and the proteins co-expressed in the serum of four groups of female mice. We performed pair comparisons with the control vs. MWCNT groups, the control vs. DBP groups, the control vs. combination groups, and the MWCNT vs. combination groups to obtain the differentially expressed proteins. The significantly expressed proteins were subjected to GO enrichment analysis, which showed that the functional localization of the significantly expressed protein was mostly focused on cell composition.

When analyzing the differential proteins in the serum of male mice, we performed the same pair comparisons as for the female mice, followed by GO enrichment analysis. We found that, similar to the serum of female mice, the functional localization of these proteins was mostly focused on cell composition. Because abundantly expressed proteins in serum affect the detection of proteins expressed in lower numbers in serum, the number of proteins detected by serum protein profiling is limited.

In the analysis of differential proteins in testicular tissues, we found that the number of detected proteins increased significantly compared to the number of proteins in the serum, and the proteins with different expression levels were also obtained by pair comparison of proteins co-expressed in the four groups. We found that the functions of the significantly expressed proteins were mostly focused on cell composition.

We analyzed the results of Venn analysis, volcano map analysis, GO enrichment analysis, and heatmap analysis. The results showed that the expression of cholesterol side-chain cleavage enzyme (encoding gene *Cyp11a1*), steroid 17-alpha-hydroxylase/17, 20 lyase (encoding gene *Cyp17a1*), 3 beta-hydroxysteroid dehydrogenase (encoding gene *Hsd3b1*), and testosterone 17-beta-dehydrogenase 3 (encoding gene *Hsd17b3*) changed significantly in the combined exposure group. Compared with the MWCNT group, the expression of 3-beta-hydroxysteroid dehydrogenase was significantly downregulated in the combined exposure group. Thus, we selected these four genes related to sex hormone synthesis to detect their expression levels.

Mitochondrial cytochrome *CYP11A1* is the only known enzyme that can cleave the side-chain of cholesterol to produce pregnenolone. Pregnenolone is the precursor of all steroid hormones, which are vital to life [42]. *CYP11A1* is one of four enzymes in vertebrates (*CYP7A1*, *CYP27A1*, *CYP46A1,* and *CYP11A1*). They initiate the biotransformation of cholesterol in different organs to maintain cholesterol homeostasis [43]. Cytochrome *CYP17* can regulate the activities of 17α-hydroxylase and 17, 20 lyase steroids. These two enzymes play an important role in the synthesis of steroid hormones [44]. Hydroxysteroid dehydrogenases (HSD3Bs and HSD17Bs) belong to the short-chain dehydrogenase/reductase family. They consist of a variety of enzymes with oxidoreductase activity. HSD17Bs can catalyze the conversion between 17-keto and 17-hydroxysteroid to affect the synthesis of steroids. The proteins encoded by genes *Cyp11a1*, *Cyp17a1*, *Hsd3b1*, and *Hsd17b3* are all involved in the synthesis of sex hormones.

When we detected the expression levels of the related genes, we found that the combined exposure in the ovary resulted in a significant decrease in the expression levels of the four genes, and the expression of *Cyp11a1*, *Cyp17a1,* and *Hsd3b1* in the combined exposure group was significantly reduced compared to the MWCNT group. We, therefore, concluded that the combination of MWCNTs and DBP reduces the rate of DBP degradation in these mice, thus increasing the effect DBP on sex hormone synthesis in female mice.

In contrast, we found that combined exposure increased the levels of *Cyp11a1*, *Cyp17a1,* and *Hsd3b1* in the testicular tissue of mice, thus affecting the sex hormone synthesis process of these mice. However, there was no significant difference in gene expression between the combined treatment group and the MWCNT group. Therefore, we can conclude that the combination of MWCNTs and DBP can affect the sex hormone synthesis in these mice, but does not increase the endocrine-disrupting effect of DBP in male mice.

## 5. Conclusions

This research focused on the fetal developmental toxicity caused by exposure to a combination of MWCNTs and DBP, which are both widely used. By evaluating the fertility of female and male mice, observing any pathological damage to the reproductive organs, and analyzing the expression of typical genes involved in the synthesis of sex hormones in the tissues, we can draw the following conclusions: DBP adsorbed on MWCNTs can cause reproductive and fetal developmental toxicity in Balb/C mice, as well as increase the fetal mortality. We found that MWCNTs as a carrier of DBP can increase the endocrine-disrupting effect of DBP by delaying the degradation of DBP in mice. Moreover, DBP affects the development of fetal by affecting the synthesis of sex hormones in female mice, increasing fetal mortality. However, we found that the male reproductive toxicity caused by DBP adsorbed on MWCNTs is not significantly related to the increase in mouse fetal mortality. In addition, we speculated that the reason why DBP adsorbed on MWCNTs increased the mortality of mouse fetuses was that it increased the content of DBP that passed through the placental barrier.

## Figures and Tables

**Table 1:** The sequences of gene primers.

Gene	Sequences of Gene Primers (5′–3′)
*Cyp11a1*	F AGGTCCTTCAATGAGATCCCTT
	R TCCCTGTAAATGGGGCCATAC
*Cyp17a1*	F GCCCAAGTCAAAGACACCTAAT
	R GTACCCAGGCGAAGAGAATAGA
*Hsd3b1*	F TGGACAAAGTATTCCGACCAGA
	R GGCACACTTGCTTGAACACAG
*Hsd17b3*	F ATGGGCAGTGATTACCGGAG
	R ACAACATTGAGTCCATGTCTGG
*β-actin*	F GGCTGTATTCCCCTCCATCG
	R CCAGTTGGTAACAATGCCATGT

**Table 2:** Fetal development statistics (n = 10); * *p* < 0.05, compared with control group.

Group (n = 10)	Counts of Live Births	Stillbirth Rate (%)
Control	3.6 ± 1.00	1.25 ± 1.25
MWCNTs	4.4 ± 1.00	2.92 ± 1.97
DBP	3.4 ± 0.99	3.65 ± 2.50
MWCNTs + DBP	4.7 ± 0.57	12.43 ± 4.61 *

## Data Availability

The datasets generated during and/or analyzed during the current study are available from the corresponding author on reasonable request.

## Supplementary Materials

The following supporting information can be downloaded at: https://www.mdpi.com/article/10.3390/toxics11070565/s1, File S1: Carbon nanotube, short multi-walled, ≥95%, OD: <8 nm, Length: 0.5–2 μm.

## References

[1] Sun C., Li W., Xu Y., Hu N., Ma J., Cao W., Sun S., Hu C., Zhao Y., Huang Q. (2020). Effects of carbon nanotubes on the toxicities of copper, cadmium and zinc toward the freshwater microalgae Scenedesmus obliquus. Aquat. Toxicol..

[2] Deng R., Yang K., Lin D. (2021). Pentachlorophenol and ciprofloxacin present dissimilar joint toxicities with carbon nanotubes to Bacillus subtilis. Environ. Pollut..

[3] Ali A., Ezz Eddin B., Chaichan M. (2021). An investigation of effect of hematocrit on thermal conductivity of a bio-nanofluid (MWCNT or SWCNT with blood). Therm. Sci. Eng. Prog..

[4] Bergamaschi E., Garzaro G., Wilson Jones G., Buglisi M., Caniglia M., Godono A., Bosio D., Fenoglio I., Guseva Canu I. (2021). Occupational Exposure to Carbon Nanotubes and Carbon Nanofibres: More Than a Cobweb. Nanomaterials.

[5] Dias J.T., Ramos G.C., Marinho P.S.B., Gester R., Andrade-Filho T. (2021). Theoretical investigation of adsorption of kojic acid on carbon nanotubes. Mater. Lett..

[6] Przybylska N., Śliwińska-Bartkowiak M., Kościński M., Rotnicki K., Bartkowiak M., Jurga S. (2021). Confined effect of water solution of ciprofloxacin in carbon nanotubes studied by Raman and Fourier Transform Infrared Spectroscopy methods. J. Mol. Liq..

[7] Kavousi M., Chavoshi M.S. (2020). Effect of coated carbon nanotubes with chitosan and cover of flaxseed in the induction of MDA-MB-231 apoptosis by analyzing the expression of Bax and Bcl-2. Meta Gene.

[8] Nayak V., Singh K.R.B., Verma R., Pandey M.D., Singh J., Pratap Singh R. (2022). Recent advancements of biogenic iron nanoparticles in cancer theranostics. Mater. Lett..

[9] Chen M., Zhou S., Zhu Y., Sun Y., Zeng G., Yang C., Xu P., Yan M., Liu Z., Zhang W. (2018). Toxicity of carbon nanomaterials to plants, animals and microbes: Recent progress from 2015-present. Chemosphere.

[10] Kobayashi N., Izumi H., Morimoto Y. (2017). Review of toxicity studies of carbon nanotubes. J. Occup. Health.

[11] Guo Y., Terazzi E., Seemann R., Fleury J.B., Baulin V.A. (2016). Direct proof of spontaneous translocation of lipid-covered hydrophobic nanoparticles through a phospholipid bilayer. Sci. Adv..

[12] Facciolà A., Visalli G., La Maestra S., Ceccarelli M., D’Aleo F., Nunnari G., Pellicanò G.F., Di Pietro A. (2019). Carbon nanotubes and central nervous system: Environmental risks, toxicological aspects and future perspectives. Environ. Toxicol. Pharmacol..

[13] Sridharan S., Taylor-Just A., Bonner J.C. (2021). Osteopontin mRNA expression by rat mesothelial cells exposed to multi-walled carbon nanotubes as a potential biomarker of chronic neoplastic transformation in vitro. Toxicol. Vitr..

[14] Albini A., Pagani A., Pulze L., Bruno A., Principi E., Congiu T., Gini E., Grimaldi A., Bassani B., De Flora S. (2015). Environmental impact of multi-wall carbon nanotubes in a novel model of exposure: Systemic distribution, macrophage accumulation, and amyloid deposition. Int. J. Nanomed..

[15] Bai Y., Zhang Y., Zhang J., Mu Q., Zhang W., Butch E.R., Snyder S.E., Yan B. (2010). Repeated administrations of carbon nanotubes in male mice cause reversible testis damage without affecting fertility. Nat. Nanotechnol..

[16] Zhang H.Y., Chen R.L., Shao Y., Wang H.L., Liu Z.G. (2018). Effects of exposure of adult mice to multi-walled carbon nanotubes on the liver lipid metabolism of their offspring. Toxicol. Res..

[17] Lü H., Mo C.H., Zhao H.M., Xiang L., Katsoyiannis A., Li Y.W., Cai Q.Y., Wong M.H. (2018). Soil contamination and sources of phthalates and its health risk in China: A review. Environ. Res..

[18] Cai Q.Y., Xiao P.Y., Zhao H.M., Lü H., Zeng Q.Y., Li Y.W., Li H., Xiang L., Mo C.H. (2017). Variation in accumulation and translocation of di-n-butyl phthalate (DBP) among rice (*Oryza sativa* L.) genotypes and selection of cultivars for low DBP exposure. Environ. Sci. Pollut. Res. Int..

[19] Cao Y., Li J., Wu R., Lin H., Lao J.-Y., Ruan Y., Zhang K., Wu J., Leung K.M.Y., Lam P.K.S. (2022). Phthalate esters in seawater and sediment of the northern South China Sea: Occurrence, distribution, and ecological risks. Sci. Total Environ..

[20] Cheshmazar E., Arfaeinia L., Vasseghian Y., Ramavandi B., Moradi M., Hashemi S.E., Asgari E., Arfaeinia H., Dragoi E.-N., Mousavi Khaneghah A. (2021). Phthalate acid esters in pickled vegetables packaged in polyethylene terephthalate container: Occurrence, migration, and estrogenic activity-associated risk assessment. J. Food Compos. Anal..

[21] Farzanehfar V., Naderi N., Kobarfard F., Faizi M. (2016). Determination of dibutyl phthalate neurobehavioral toxicity in mice. Food Chem. Toxicol..

[22] Zhu Q., Xu L., Wang W., Liu W., Liao C., Jiang G. (2022). Occurrence, spatial distribution and ecological risk assessment of phthalate esters in water, soil and sediment from Yangtze River Delta, China. Sci. Total Environ..

[23] Zhu Y.D., Han X., Wang X.Q., Ge T.X., Liu H., Fan L., Li L., Su L.Q., Wang X.L. (2022). Effect of the phthalates exposure on sex steroid hormones in the US population. Ecotoxicol. Environ. Saf..

[24] Silinski M.A.R., Fernando R.A., Robinson V.G., Waidyanatha S. (2020). Development and Validation of an Analytical Method for Quantitation of Monobutylphthalate, a Metabolite of Di-n-Butylphthalate, in Rat Plasma, Amniotic Fluid, Fetuses and Pups by UPLC-MS/MS. J. Anal. Toxicol..

[25] Jauregui E., Lock J., Rasmussen L., Craig Z. (2021). Mono-n-butyl phthalate distributes to the mouse ovary, liver and alters the expression of phthalate-metabolizing enzymes in both tissues. Toxicol. Sci..

[26] Maestre-Batlle D., Pena O.M., Huff R.D., Randhawa A., Carlsten C., Bølling A.K. (2018). Dibutyl phthalate modulates phenotype of granulocytes in human blood in response to inflammatory stimuli. Toxicol. Lett..

[27] Matthew S., Nwannenna A., Samuel F., Rekwot P.I. (2020). Melatonin and garlic cytoprotective-ameliorative effects on dibutyl phthalate intoxication on sperm DNA and testicular biomakers of rabbits. Sokoto J. Vet. Sci..

[28] Liu X., Craig Z.R. (2019). Environmentally relevant exposure to dibutyl phthalate disrupts DNA damage repair gene expression in the mouse ovary. Biol. Reprod..

[29] Wu N., Tao L., Tian K., Wang X., He C., An S., Tian Y., Liu X., Chen W., Zhang H. (2023). Risk assessment and environmental determinants of urinary phthalate metabolites in pregnant women in Southwest China. Environ. Sci. Pollut. Res..

[30] He X., Zang J., Liao P., Zheng Y., Lu Y., Zhu Z., Shi Y., Wang W. (2019). Distribution and Dietary Predictors of Urinary Phthalate Metabolites among Pregnant Women in Shanghai, China. Int. J. Environ. Res. Public Health.

[31] Badea N., Craciun M.M., Dragomir A.S., Balas M., Dinischiotu A., Nistor C., Gavan C., Ionita D. (2020). Systems based on carbon nanotubes with potential in cancer therapy. Mater. Chem. Phys..

[32] Faraji Dizaji B., Khoshbakht S., Farboudi A., Azarbaijan M.H., Irani M. (2020). Far-reaching advances in the role of carbon nanotubes in cancer therapy. Life Sci..

[33] Dutt M.A., Hanif M.A., Nadeem F., Bhatti H.N. (2020). A review of advances in engineered composite materials popular for wastewater treatment. J. Environ. Chem. Eng..

[34] Wang F., Yao J., Sun K., Xing B. (2010). Adsorption of dialkyl phthalate esters on carbon nanotubes. Environ. Sci. Technol..

[35] Zhou T., He Y., Qin Y., Wang B., Zhang H., Ding S. (2022). Exposure to a combination of MWCNTs and DBP causes splenic toxicity in mice. Toxicology.

[36] Barbăroșie C., Agarwal A., Henkel R. (2021). Diagnostic value of advanced semen analysis in evaluation of male infertility. Andrologia.

[37] Priya K., Setty M., Babu U.V., Pai K.S.R. (2021). Implications of environmental toxicants on ovarian follicles: How it can adversely affect the female fertility?. Environ. Sci. Pollut. Res..

[38] Czubacka E., Czerczak S., Kupczewska-Dobecka M.M. (2021). The overview of current evidence on the reproductive toxicity of dibutyl phthalate. Int. J. Occup. Med. Environ. Health.

[39] Ahangarpour A., Alboghobeish S., Oroojan A.A., Dehghani M.A. (2021). Caffeic acid protects mice pancreatic islets from oxidative stress induced by multi-walled carbon nanotubes (MWCNTs). Vet. Res. Forum.

[40] Takeshima T., Usui K., Mori K., Asai T., Yasuda K., Kuroda S., Yumura Y. (2021). Oxidative stress and male infertility. Reprod. Med. Biol..

[41] Wu P.Y., Scarlata E., O’Flaherty C. (2020). Long-Term Adverse Effects of Oxidative Stress on Rat Epididymis and Spermatozoa. Antioxidants.

[42] Kaur R., Kaur T., Sudhir N., Kaur A. (2021). Association Analysis of CYP11A1 Variants with Polycystic Ovary Syndrome: A Case-Control Study from North India. Reprod. Sci..

[43] Mast N., Annalora A.J., Lodowski D.T., Palczewski K., Stout C.D., Pikuleva I.A. (2011). Structural Basis for Three-step Sequential Catalysis by the Cholesterol Side Chain Cleavage Enzyme CYP11A1. J. Biol. Chem..

[44] Xu X., Hu K., Shi H., Yu Y., Xu J., Sun Y. (2021). The single-nucleotide polymorphism rs743572 of CYP17A1 shows significant association with polycystic ovary syndrome: A meta-analysis. Reprod. Biomed. Online.

