# Supplementary material for: Dibutyl Phthalate Adsorbed on Multiwalled Carbon Nanotubes Causes Fetal Developmental Toxicity in Balb/C Mice
Source: Toxics. 2023 Jun 29;11(7):565. doi: 10.3390/toxics11070565 (PMC10385951; doi:10.3390/toxics11070565)
Supplement: Supplementary file 1 [file toxics-11-00565-s001.zip › toxics-2415830-supplementary.pdf]

No. 196 xinjinqiao Road, Pudong District, Shanghai 201206, China
TELEPHONE : 400 620 6333 FAX : +86-21 5032 3701
TECH EMAIL : tech@aladdin-e.com

CERTIFICATE OF ANALYSIS

PRODUCT NAME: Carbon nanotube, short multi-walled,≥95%,OD:＜8nm,Length:0.5－2um

ITEM NUMBER: C140996
LOT NUMBER: H1810165
BRAND: Aladdin
CAS NUMBER: 308068-56-6
MDL NUMBER:
FORMULA:
MOLECULAR WEIGHT:
QUALITY RELEASE DATE: 2018-08-16 18:34:35
RECOMMENDED RETESTED DATE: 2022-08-15 18:34:35

TEST	SPECIFICATION MIN.	SPECIFICATION MAX.	TARGET VALUE	RESULT
Appearance	black powder			black powder
ash	0 %	1.5 %	1.5 %	1.230000 %
Diameter	0 nm	8 nm	8 nm	5.680000 nm
Length	0.5 μm	2 μm	2 μm	1.620000 μm
Purity	95 %	100 %	100 %	95.000000 %
Specific surface area	0 m2/gsurface	0 m2/gsurface	>350 m2/gsurface	361.000000 m2/gsurface

Julian Xu
Shanghai ALADDIN Biochemical Technology Co.Ltd

Aladdin warrants, that at the time of the quality release or subsequent retest date this product conformed to the information contained in this publication. The current Specification sheet may be available at www.aladdin-e.com. For further inquiries, please contact Technical Service. Purchaser must determine the suitability of the product for its particular use. See reverse side of invoice or packing slip for additional terms and conditions of sale

VERSION NUMBER: 1
